# Culture as a catalyst for regenerative co-creation: the role of yuanfen in driving customer voice through social bonds in P2P accommodation

**DOI:** 10.3389/fpsyg.2026.1759683

**Published:** 2026-03-10

**Authors:** Huifang Liu, Xiaoting Huang

**Affiliations:** 1 School of Management, Shandong University, Jinan, China; 2 School of Tourism, Xinjiang College of Science and Technology, Korla, China

**Keywords:** P2P accommodations, regenerative co-creation, yuanfen, social bonds, perceived customer effectiveness, customer voice intention

## Abstract

**Introduction:**

As tourism shifts from sustainability toward regeneration, active customer participation has become essential for revitalizing the peer-to-peer (P2P) accommodation sector. However, the cultural values that motivate such participation remain largely overlooked. Drawing on Cognitive Appraisal Theory, this study investigates how yuanfen, a culturally embedded attributional appraisal of predestined connectedness, shapes customer voice intention in P2P accommodation contexts.

**Methods:**

This study employed a scenario-based experiment manipulating interpersonal and physical-environmental cues. Data were collected through a structured questionnaire, and structural equation modeling (SEM) was used to test the hypothesized relationships among perceived yuanfen, social bonds, perceived customer effectiveness (PCE), and customer voice intention.

**Results:**

Both interpersonal and physical-environmental cues were found to effectively evoke perceived yuanfen. Higher levels of perceived yuanfen significantly increased customer voice intention, with social bonds acting as a key mediating mechanism. Moreover, the moderating analysis indicated that when perceived customer effectiveness (PCE) is low, the influence of social bonds on voice intention becomes stronger, identifying boundary conditions under which relational motivations are most behaviorally consequential.

**Discussion:**

These findings demonstrate how culturally embedded appraisals such as yuanfen can shape prosocial engagement through strengthened social bonds. The results contribute to understanding how cultural meaning-making processes influence customer voice within P2P accommodation settings. Practically, P2P platforms and hosts may encourage constructive feedback by incorporating interpersonal and environmental cues that evoke relational warmth and connectedness.

## 1 Introduction

Customer voice has long been recognized as a valuable resource for organizations, providing actionable suggestions that enhance service quality and stimulate innovation (Efe et al., 2025b). Many firms, such as LEGO and Starbucks, actively encourage customers to participate in co-creation processes in order to generate new ideas and strengthen competitiveness (Wong et al., 2016). In the context of peer-to-peer (P2P) accommodation, customer voice has become increasingly relevant as the industry continues to expand (Chen et al., 2023; Yang et al., 2024; Jiang and Chen, 2025). Unlike traditional hospitality settings, most P2P accommodations are operated by self-employed micro-entrepreneurs (Zhang et al., 2019), who often face financial pressure and heightened vulnerability to market fluctuations (Wang J. et al., 2025). These pressures may translate into emotional challenges, including anxiety and loneliness (Zhang et al., 2023b; Tao et al., 2024). Within such relationally intensive service settings, customer voice may extend beyond functional exchanges by strengthening relational ties, encouraging repeat patronage (Eisingerich et al., 2014), supporting service innovation (Lee et al., 2019) and fostering trust between hosts and guests (Tao et al., 2024). At a broader level, repeated host–guest interactions and the intimacy of B&B encounters may accumulate into trust-building micro-relations that function as foundational social units within the wider tourism ecosystem (Pera et al., 2019), facilitating meaningful and reciprocal engagement among tourism stakeholders (Iddawala and Lee, 2025). Despite its potential importance, research has paid comparatively limited attention to customer voice and its underlying antecedents in P2P accommodation contexts (Wan and Li, 2021; Efe et al., 2025b).

The conceptualization of customer voice as a discretionary, extra-role behavior draws on the broader tradition of employee voice in organizational behavior research, while also extending into service and marketing domains. However, its definition has not reached a clear consensus in the literature. Some studies conceptualize customer voice primarily as customer complaints (Bove and Robertson, 2005; Lacey, 2012; Osakwe et al., 2024), whereas others conceptualize it as voluntary suggestions that support organizational development (Ran and Zhou, 2020; Chen and Li, 2021; Yang et al., 2021 Yang et al., 2021). A third stream views customer voice as a combination of complaints and constructive suggestions (Hibbard et al., 2001; Assaf et al., 2015; Efe et al., 2025a). While customer complaint behavior has been examined extensively since the 1970s (Nasr et al., 2014), understanding customer voice as a discretionary and proactive behavior has become increasingly important in dynamic service environments. Consistent with recent research, this study defines customer voice as voluntary communication through which customers provide constructive suggestions to businesses (Ran and Zhou, 2020).

Existing research has primarily focused on relational and managerial antecedents of customer voice, including customer–employee interactions (Chen and Li, 2021), relationship quality (Ran and Zhou, 2019, 2020), membership management practices (Béal and Sabadie, 2018), and online community management (Yang et al., 2021; Liao et al., 2024). By contrast, the cultural foundations underlying customer voice behavior remain underexplored. Yuanfen (缘分), a culturally embedded belief widely shared in Chinese society, represents one such overlooked influence. Although emerging research has begun to examine yuanfen in service contexts, empirical investigations remain limited (Tang and Fong, 2024).

Rooted deeply in Chinese cultural traditions (Yau, 1994), yuanfen reflects a culturally shaped cognitive appraisal through which individuals interpret relational encounters as meaningful and fated. In everyday Chinese interactions, individuals frequently interpret unexpected meetings through this appraisal lens, which has been shown to shape subsequent relational and emotional responses. Such appraisals have been shown to reduce psychological distance, foster emotional closeness, and encourage prosocial relational responses, including revisiting destinations or recommending hosts (Jiang et al., 2022; Tang and Fong, 2024). Beyond direct interpersonal interaction, symbolic and physical elements within service environments may also function as culturally meaningful cues that trigger fate-based appraisals. In P2P accommodation settings, supportive design features and humanistic atmospheres may extend the application of yuanfen beyond person–person relationships to person–object encounters (Tang and Fong, 2024; Wang S. et al., 2025).

Although prior studies have linked yuanfen to host–guest and guest–guest relationships (Zhang et al., 2020; Tang et al., 2024), existing scholarship relies heavily on qualitative insights and lacks quantitative evidence regarding its influence on specific service outcomes such as proactive engagement or voice behavior (Tang and Fong, 2024). Moreover, research has predominantly emphasized interpersonal dimensions, leaving the potential role of yuanfen in person–object contexts largely unexplored. This gap limits theoretical development as well as practical guidance for platform design and host strategies (Tang and Fong, 2024). In P2P accomodations, physical artifacts and atmospheric designs are often extensions of the host’s identity (Wang S. et al., 2025), enabling guests to form meaningful connections not only with the host but also with the environment itself. These object-related cues may activate culturally grounded appraisals of yuanfen, shaping customer engagement in interaction-intensive service encounters (Chang and Holt, 1991). Understanding how these culturally embedded appraisals shape customers’ voice intentions is therefore an important research direction.

To bridge these gaps, this study employs a scenario-based experimental design to investigate whether and how perceived yuanfen influences customers’ voice intentions in P2P accommodation. We examine yuanfen across both interpersonal (person–person) and person–object contexts. Specifically, two sets of experimental stimuli are developed: the first manipulates perceived interpersonal affinity with the host, while the second captures guests’ perceived connection to symbolic and physical elements of the accommodation environment. Focusing on customers’ intended voice responses, we systematically test the psychological mechanism through which yuanfen perception promotes voice intention via social bonds, and further explore the moderating role of customers’ perceived effectiveness in this process.

This study makes several key contributions. First, drawing on Cognitive Appraisal Theory, it conceptualizes yuanfen as a culturally shaped appraisal that strengthens social bonds and subsequently promotes customers’ voice intentions in P2P accommodation. Second, it reveals a counterintuitive moderating effect of perceived customer effectiveness, showing that the influence of social bonds on voice intention becomes stronger when customers feel less effective. Finally, by examining yuanfen across both interpersonal and person–object contexts, this research broadens the conceptual scope of yuanfen in service encounters and provides practical implications for fostering constructive customer participation in sharing-based platforms.

## 2 Literature review

### 2.1 Cognitive appraisal theory

Cognitive appraisal theory posits that individuals’ behavioral responses arise from cognitive evaluations of events or stimuli in relation to their personal goals, motives, and well-being (Smith and Ellsworth, 1985; Smith and Lazarus, 1993). This process involves two sequential stages. In primary appraisal, individuals assess the personal relevance and significance of an encounter. In secondary appraisal, they evaluate their available coping resources and perceived efficacy in managing the situation (Smith and Lazarus, 1993). Positive primary appraisals often generate motivational tendencies that facilitate goal-directed actions, whereas secondary appraisals concerning coping effectiveness may strengthen or constrain the translation of these motivations into behavioral intentions.

CAT has been widely applied in tourism and hospitality research to explain how tourists and customers interpret service encounters and develop subsequent relational and behavioral responses (Choi and Choi, 2019). For instance, in heritage and destination contexts, perceived authenticity or historical relevance triggers primary appraisals of existential or interpersonal significance, eliciting relational motives that mediate protective or cohesive behaviors (Meng and Luo, 2023). Similarly, anthropomorphic communication in hotel settings elicits cognitive appraisals of service attributes such as perceived coolness, warmth, and cuteness, which in turn enhance relational brand attachment and customers’ willingness to pay premium prices (Li et al., 2023).

### 2.2 Yuanfen

Yuanfen is a culturally embedded concept deeply rooted in Chinese social life (Tang et al., 2024). In this study, yuanfen is conceptualized as a culturally grounded attributional appraisal through which individuals interpret encounters as meaningful or predestined. Rather than representing a transient emotion, yuanfen functions as an interpretive lens that allows individuals to attribute relational encounters to an external and fate-related cause. Yuanfen-related encounters are often perceived as low-probability yet meaningful occurrences (Yang, 1998), reflecting a culturally shaped mode of understanding why relationships emerge and how they evolve.

Historically, yuanfen draws from Buddhist notions of interconnected causes and conditions and karmic causality, later shaped by Confucian relational ethics that emphasize social harmony. Within this cultural tradition, attributing encounters to yuanfen serves as a socially accepted explanatory mechanism that helps individuals retrospectively make sense of relational outcomes in a complex social environment (Chang and Holt, 1991). As an external attribution, yuanfen can be used to interpret both favorable and unfavorable relationship outcomes. For instance, attributing positive relationships (e.g., a satisfying marriage) to yuanfen rather than to personal effort may reduce envy and sustain social harmony (Yang and Ho, 1988). Similarly, by framing unchangeable adversities as fated, yuanfen facilitates acceptance and offers psychological comfort and emotional relief (Hsu and Hwang, 2016). Although yuanfen may be invoked in negative contexts, contemporary discourse more often associates it with positive and meaningful encounters (Tang and Fong, 2024). In tourism and hospitality research, prior studies have linked yuanfen to host–guest and guest–guest relationships (Zhang et al., 2020), showing that yuanfen-related ties—such as friendships formed with fellow travelers—are perceived as positive tourism experiences (Wang and Xie, 2021) and and satisfying accommodation experiences (Zhang et al., 2020).

Over time, everyday discourse has broadened the interpretive application of yuanfen beyond purely interpersonal relationships. Rather than being confined to explaining person–person ties, yuanfen is increasingly invoked to make sense of connections involving places, objects, and events. Within interpersonal contexts, people commonly refer to culturally specific expressions of yuanfen (e.g., kinship yuanfen, marital yuanfen, academic yuanfen, geographic yuanfen, occupational yuanfen, and financial yuanfen), highlighting its explanatory function across diverse relationship domains such as kinship, marriage, romance, and friendship. More recently, yuanfen has also been used to interpret perceived connections between individuals and destinations, environments, or symbolic objects (Zhang et al., 2020; Jiang et al., 2022; Tang and Fong, 2024; Tang et al., 2024). For instance, Jiang et al. (2022) found that yuanfen-based connections among strangers may facilitate tourists’ tendency to attribute their experiences at a destination to yuanfen.

People’ s understanding of the properties of a given attribution shapes their responses, including emotional and behavioral reactions (Weiner, 2014). Accordingly, grounded in attribution theory, attributing an encounter to yuanfen implies the perception of an intangible bond between the parties involved. This perceived bond can reduce psychological distance (Yi and Chen, 2015), fostering a sense of closeness that may be subjectively experienced as a “mind-to-mind connection.” Such attributional appraisals may elicit specific emotional responses (e.g., psychological closeness, gratitude, and warmth; see Table 1) and, in turn, encourage positive behavioral tendencies. In narratives where encounters are attributed to yuanfen, individuals commonly express intentions to return, recommend, and stay longer (Tang et al., 2024). Consistent with these tendencies, yuanfen-related interpretations have also been associated with higher hotel ratings (Tang and Fong, 2024) and increase tolerance for service failures (deMatos et al., 2021).

**Table 1:** Conceptual distinctions between yuanfen and related constructs.

Source	Construct	Definition	Core characteristics
Tang and Fong (2024)	Yuanfen	It reflects a culturally shaped cognitive appraisal through which individuals interpret relational encounters as meaningful and, to some extent, fated.	•Cognitive attribution • External, fate-related attribution • Situation-activated
Fang et al. (2023)	Social bond	Customers’ perceived relational orientation and willingness to establish and maintain a connection.	• Relational state • Conative (willingness)
Park et al. (2025) and Liu et al. (2026)	Emotional attachment	A deep, enduring affective bond with a target, characterized by emotional reliance and integration.	• Enduring affect • Requires time/history • High intensity
Jiang and Chen (2025) and Nguyen and Yang (2025)	Gratitude	As a trait, a generalized tendency to feel thankful; as a state, an emotional response to perceived benevolence or kindness from others.	• Reciprocity-based • Triggered by benefit • Moral obligation
Carr and Rosaen (2024) and Tang and Fong (2024)	Relational closeness	Subjective perception of psychological proximity and interpersonal warmth.	• Subjective perception • Psychological distance • Dyadic focus
Ren et al. (2018) and Peng et al. (2026)	Warmth	A positive, mild, and volatile affective response in interactions.	• Immediate affect • Physiological/Emotional • Volatile (fleeting)

### 2.3 Yuanfen and customers’ voice intention

The study of voice behavior originates from Hirschman’s (1970) Exit, Voice, and Loyalty framework, which posits that when satisfaction declines, individuals respond by either voicing their concerns or exiting the relationship. Employees with stronger loyalty are more inclined to express their opinions rather than withdraw. LePine and Van Dyne (1998) introduced the concept into organizational behavior research, prompting extensive investigations into employee voice. More recently, this concept has been extended to customer contexts, where customer voice is understood as proactive feedback and voluntary suggestions that contribute to service improvement and relationship maintenance (Ran and Zhou, 2020; Chen and Li, 2021; Yang et al., 2021; Liao et al., 2024).

Customer voice differs from complaints in that it reflects constructive and future-oriented input (Bove et al., 2009). By providing suggestions, reporting concerns, or offering constructive criticism, customers help firms enhance service quality and operational effectiveness (Gong and Choi, 2016; Assaf et al., 2022). Nasr et al. (2014) identified five forms of customer feedback—the “5Cs”: compliments, complaints, comments (neutral descriptions of product use), concerns (issues that could become problems if unaddressed), and counsel (suggestions and constructive criticism). The latter two are generally regarded as manifestations of customer voice. In the P2P accommodation, where hosts are often vulnerable micro-entrepreneurs, customer voice may carry additional relational significance. Following Oertzen et al. (2018), we conceptualize customer voice in this setting as an act of regenerative co-creation that renews and sustains the service ecosystem. Unlike general customer citizenship behavior, which may occur between tourists or between tourists and potential travelers, voice in P2P accommodation occurs specifically within the host–guest dyad. This host–guest interaction actively (re)builds positive relationships between tourists and the destination, thereby promoting the regenerative development of the tourism community from a social dimension (Vegas-Macias et al., 2026). Guests not only provide economic support to hosts but also, through voice as an extra-role behavior, offer guidance in service performance while providing emotional support and altruistic care. A case study shows that host–guest trust relationships can persist long after the trip ends and may even transform into trust capital, enabling guests to provide support and assistance when hosts need help in the future (Pang and Sun, 2025).

Yuanfen differs from classical attribution theory (Heider, 1958), which explains behavior through both internal (personal) and external (environmental) causes. In many Chinese cultural contexts, however, encounters are often interpreted through fate-related attributions that emphasize forces beyond individual control (Norenzayan and Lee, 2010). Consistent with this orientation, perceived yuanfen can be understood as a culturally embedded attributional appraisal through which individuals interpret an encounter as meaningful and, to some extent, predestined, rather than as the outcome of purely rational causal reasoning. Empirical research suggests that individuals’ perceived yuanfen with a hotel is associated with higher rating behavior (Tang and Fong, 2024) and stronger brand preference for similar products (Yang and Song, 2024). Beyond economic outcomes, yuanfen-related interpretations have been linked to greater tolerance toward service failures (deMatos et al., 2021) andincreased likelihood of recommendation and positive word-of-mouth (Tang et al., 2024).

Drawing from CAT, such culturally shaped appraisals may influence subsequent emotional and behavioral responses. In P2P accommodation settings, where hosts often engage directly with guests, service encounters may be interpreted through the lens of yuanfen. When individuals perceive yuanfen in host–guest interactions, this attributional appraisal of meaningful connection may increase their willingness to offer constructive suggestions, positioning customer voice as a relationally supportive behavior rather than merely transactional feedback. Although customer voice does not in itself constitute regenerative co-creation, it may function as a micro-level relational mechanism that supports mutual adaptation within host–guest relationships. Accordingly, this study proposes the following hypothesis:

*H1*: Customers with higher (vs. lower) perceived yuanfen are more likely to report stronger voice intention.

### 2.4 The mediating role of social bonds

Social bonds refer to the social and emotional connections formed between individuals through interaction and mutual understanding (Jonsson and Zineldin, 2003; Bellocchi, 2022). They reflect relational closeness, affection, and willingness to develop interpersonal ties (Wilson, 1995). In service contexts, social bonds may emerge not only through direct interpersonal encounters but also through symbolic and experiential cues that convey warmth and care (Pang et al., 2024). In this study, social bonds denote guests’ sense of relational closeness and their willingness to maintain a connection with the host. In P2P accommodations, even limited interaction can foster such bonds through tangible cues, such as welcome gifts or personalized notes, that convey the host’s attentiveness and care (Farmaki et al., 2020). Moreover, Similarly, online communication and engagement with in-room elements can evoke pleasant surprise and a sense of closeness (Zhang et al., 2025).

From a cultural perspective, Chinese guests may interpret host–guest encounters through the lens of yuanfen, perceiving the relationship as meaningful and fate-related. Cultural design elements and interaction cues may therefore be interpreted as signals consistent with yuanfen, enhancing guests’ perception of relational compatibility. When such appraisals occur, guests may become more motivated to value and sustain the relationship. Self-expansion theory suggests that individuals who perceive meaningful connections are motivated to incorporate others into their self-concept and invest in relationship development (Aron and Aron, 1996; Park et al., 2010). Accordingly, when guests perceive yuanfen and develop cultural identification with a P2P accommodation, they are more likely to form social bonds with the host, which motivate proactive engagement, such as providing feedback and suggestions aimed at improving the accommodation.

Drawing on CAT, perceived yuanfen represents a culturally embedded evaluation that gives meaning to the lodging experience. Such appraisals can elicit relationally oriented motivations, reflected in the formation of social bonds. In turn, social bonds translate yuanfen-based appraisals into relationship-supportive behaviors, such as customer voice, which requires personal involvement and investment. Therefore, we propose that social bonds mediate the relationship between perceived yuanfen and customers’ voice intention.

*H2*: Social bonds mediate the relationship between perceived yuanfen and customers’ voice intention.

### 2.5 Perceived customer effectiveness

Self-efficacy is not a fixed trait but a dynamic belief shaped by situational contexts, social influences, and affective states (Bandura, 1977). In consumer research, Perceived Customer Effectiveness (PCE) extends this concept, defined as the belief that one’ s personal actions can make a meaningful difference to outcomes or the environment (Ellen et al., 1991; Lavuri, 2022; Nguyen and Yang, 2025). In pro-environmental research, green self-efficacy has been identified as a key determinant of pro-environmental behaviors, such as green consumption and eco-friendly travel (Jaiswal and Kant, 2018; Liu-Lastres et al., 2025). Individuals with higher PCE better understand how their behaviors relate to environmental sustainability. They are also more willing to act responsibly, such as buying eco-friendly products or choosing green accommodations (Yan and Chai, 2021; Kumar et al., 2022). In the context of P2P accommodation, PCE reflects a customer’s confidence that their voice or suggestions will be influential and valued by the host (Nguyen and Chiu, 2023). Conversely, consumers with low PCE may perceive their input as negligible, leading to silence even when they are satisfied.

From the perspective of CAT, PCE functions as a secondary appraisal, specifically assessing coping potential (Smith and Lazarus, 1993). While primary appraisal (e.g., perceiving yuanfen) determines the emotional significance of an encounter, secondary appraisal evaluates the individual’s agency to manage or influence that encounter (Choi and Choi, 2019). Traditional applications of CAT suggest a synergistic relationship: high coping potential amplifies emotional pathways to action. When individuals believe they possess the capability to contribute meaningfully (high PCE), their emotional motivations are more likely to translate into active behaviors (Liu et al., 2024).

Building on this framework, we propose that PCE acts as a critical boundary condition for the impact of social bonds on voice intention. Yuanfen-based social bonds provide the affective motivation, while PCE provides the cognitive confidence. We argue that these two forces should operate synergistically to drive customer voice. Specifically, when customers feel a strong social bond and believe their feedback is effective (high PCE), they should exhibit the highest intention to voice, as they feel both the relational obligation and the functional capacity to help. In contrast, under low PCE, customers may feel that their efforts would be futile or unappreciated; this lack of efficacy may inhibit the translation of relational warmth into constructive feedback, thereby weakening the effect of social bonds. Based on this, the following hypothesis is proposed:

*H3*: Perceived customer effectiveness moderates the relationship between social bonds and customers’ voice intention, such that the effect of social bonds on voice intention is stronger (weaker) when perceived customer effectiveness is high (low).

**Figure 1:**
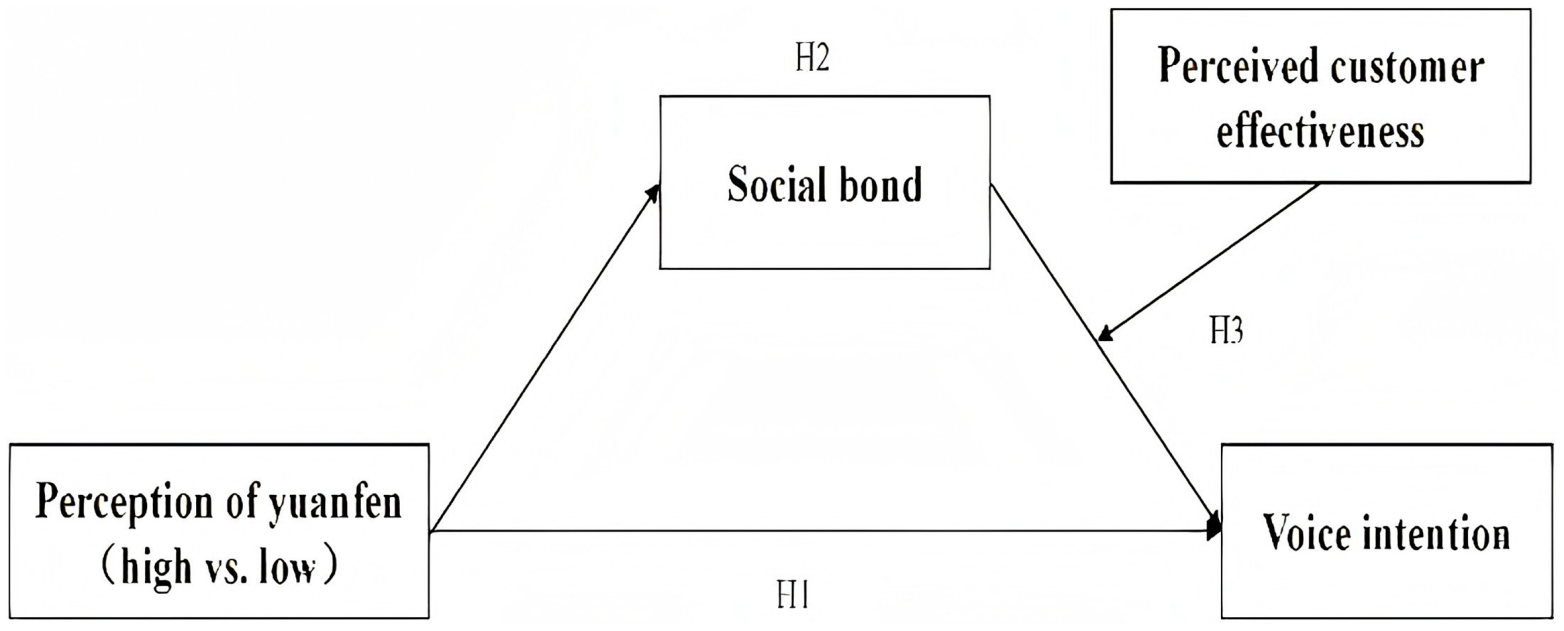
Hypothetical model.

To test the hypotheses, we developed a conceptual model (Figure 1) and conducted three experiments to examine the main, mediating, and moderating effects. Two sets of stimuli were designed to test the effects of person–person yuanfen perception and person–object yuanfen perception on customer voice intention. All experiments were conducted in China using Chinese materials, and data were collected through the Credamo platform. In tourism research, Credamo has been widely used, demonstrating its strong applicability and reliability (Su et al., 2025). As the original scales were developed in English, they were translated by two bilingual scholars and back-translated to ensure accuracy and conceptual equivalence.

## 3 Study 1

### 3.1 Pretest text-based stimuli

#### 3.1.1 Experimental design and procedure

Study 1 examined the effect of customers’ perceived yuanfen on their evaluations of P2P accommodation (H1) and tested whether yuanfen perception could be experimentally elicited. A single-factor between-subjects design was employed (yuanfen perception: high vs. low).

Participants imagined working in a distant city and choosing a rural P2P accommodation for a short holiday. They were randomly assigned to one of two scenarios. In the high-yuanfen condition, the host shared the participant’s hometown and favorite dishes, creating a sense of connection and shared memories. In the low-yuanfen condition, the interaction remained brief and impersonal, showing no shared background or emotional resonance (see Appendix A).

A total of 60 participants were recruited for the pretest. An online questionnaire containing the scenario materials was randomly distributed. After reading the materials, participants completed a manipulation check using a single-item measure adapted from prior studies. On a seven-point Likert scale (1 = strongly disagree, 7 = strongly agree), they rated: “I feel that I have yuanfen with this P2P accommodation host.” Higher scores indicated stronger perceived yuanfen toward the P2P accommodation.

#### 3.1.2 Results

A total of 60 valid responses were collected, with 30 participants in each condition. An independent-samples t-test showed that perceived yuanfen was significantly higher in the high-yuanfen condition (*M*_high_ = 6.33, *SD* = 0.71) than in the low-yuanfen condition (*M*_low_ = 3.60, *SD* = 1.40), t(58) = −9.51, *p* < 0.001, confirming the success of the manipulation for use in the main experiment.

### 3.2 Pretest key card-based stimuli

#### 3.2.1 Experimental design and procedure

Building on the preliminary study, this experiment replaced the textual stimuli with visual cues to further examine how yuanfen perception influences customers’ voice intention. A single-factor between-subjects design was employed (yuanfen perception: high vs. low). A total of 30 participants were recruited for the pretest.

To manipulate yuanfen perception, P2P accommodation key card with yuanfen-themed designs were created. Although yuanfen is often regarded as a natural coincidence, it can also serve as a symbolic nudge (Tang and Fong, 2024). Following Tang and Fong (2024), the high-yuanfen card featured symbolic text: “We are destined to meet though miles apart. Among so many places, your choice of ours is yuanfen—a fortunate encounter.” The low-yuanfen card contained only factual details: “H P2P accommodation. Tel: 0972xxxx. Address: No. 29 Renmin Road. Welcome.”

Participants were randomly assigned to either the high-yuanfen or low-yuanfen condition. After viewing the experimental materials, they completed the manipulation check, mediator, and dependent variable (customers’ voice intention). Following prior studies, a single-item measure was used for the manipulation check. On a seven-point Likert scale (1 = strongly disagree, 7 = strongly agree), participants rated: “I feel that I have yuanfen with this P2P accommodation.” Higher scores indicated stronger perceived yuanfen toward the P2P accommodation.

#### 3.2.2 Results

A total of 30 valid responses were collected, with 15 participants in each condition. An independent-samples t-test showed that perceived yuanfen was significantly higher in the high-yuanfen image condition (*M*_high_ = 5.80, *SD* = 0.78) than in the low-yuanfen image condition (*M*_low_ = 5.07, *SD* = 0.96), t(28) = −2.30, *p* = 0.029. This confirms that the manipulation of yuanfen images was successful for use in the main experiment.

### 3.3 Main experiment

#### 3.3.1 Experimental design and procedure

In the main experiment of Study 1, textual stimuli were used because the pretest showed that the text-based materials produced a clearer differentiation effect. A single-factor between-subjects design was employed (yuanfen perception: high vs. low), with participants randomly assigned to either the high-yuanfen (*n* = 65) or low-yuanfen (*n* = 65) condition. Before the experiment, participants read an instruction asking them to imagine working in a city far from home and spending a relaxing holiday with friends in a nearby rural area (S Village), where they chose a local P2P accommodation. They were then exposed to the corresponding scenario materials (see Appendix A for details).

After reading the scenario materials, participants completed the manipulation check. A single-item measure from prior studies was used. On a seven-point Likert scale (1 = strongly disagree, 7 = strongly agree), participants rated: “I feel that I have yuanfen with this P2P accommodation host.” Higher scores indicated stronger perceived yuanfen.

Participants then answered questions on customers’ voice intention and demographics (gender, age, monthly income, education, and occupation). The frequency of P2P accommodation stays in the past year was included as a control variable.

Voice intention was measured using a four-item scale adapted from Bove et al. (2009). Sample items were: “I would suggest ways for the host to improve the service” and “I would let the host know how to better meet my needs.” All items were rated on a seven-point Likert scale (1 = strongly disagree, 7 = strongly agree), with higher scores indicating stronger voice intention.

#### 3.3.2 Results

A total of 130 valid responses were collected (63.8% female, 36.2% male). Participants were randomly assigned to the high-yuanfen (*n* = 65) or low-yuanfen (*n* = 65) condition.

An independent-samples t-test revealed that participants in the high-yuanfen condition reported significantly higher perceived yuanfen (M_high_ = 6.18, SD = 0.73) than those in the low-yuanfen condition (M_low_ = 3.11, SD = 1.23), *t*(104.00) = −17.40, *p* < 0.001. These results confirms the success of the manipulation.

The voice intention scale demonstrated excellent reliability (*α* = 0.90; M = 5.40, SD = 1.08). Results showed that participants in the high-yuanfen condition reported significantly higher voice intention (M_high_ = 5.76, SD = 0.72) than those in the low-yuanfen condition (M_low_ = 5.05, SD = 1.25), *t*(101.95) = −4.01, *p* < 0.001. After controlling for gender, age, occupation, education, monthly income, and P2P accommodation stay frequency, an ANOVA indicated that the effect of yuanfen perception on voice intention remained significant, *F*(1, 122) = 16.52, *p* < 0.001, η^2^ = 0.12.

Additionally, the frequency of P2P accommodation stays had a significant effect on voice intention (*F*(1, 122) = 4.98, *p* < 0.05, η^2^ = 0.04), while the effects of other control variables were not significant. Hypothesis 1 was supported.

## 4 Study 2

### 4.1 Study 2a text-based stimuli

#### 4.1.1 Experimental design and procedure

Building on the preliminary studies, this experiment examined the mechanism through which yuanfen perception affects customers’ voice intention. A single-factor between-subjects design was employed (yuanfen perception: high vs. low), using textual stimuli. Social bond was included as a mediator. A total of 170 participants were recruited, yielding 170 valid responses.

Participants were randomly assigned to either the high-yuanfen or low-yuanfen condition. After reading the experimental materials (the same as in Study 1), they completed the manipulation check, social bond, and customers’ voice intention measures in sequence.

The manipulation check followed the same procedure as in previous studies. Participants then completed measures of social bond, voice intention, and demographic information (gender, age, monthly income, education level, and occupation). The frequency of P2P accommodation stays in the past year was included as a control variable.

The social bond scale was adapted from prior research and consisted of three items (e.g., “I would like to interact directly with the host” Fang et al., 2023). The customers’ voice intention scale was also adapted from previous studies and included four items (e.g., “I would let the host know of ways that could better serve my needs” Bove et al., 2009). All items were measured on a seven-point Likert scale (1 = strongly disagree, 7 = strongly agree), with higher scores indicating stronger social bond or voice intention.

#### 4.1.2 Results

A total of 170 questionnaires were collected. After excluding invalid responses, 166 valid samples remained (58.4% female, 41.6% male). Participants were randomly assigned to the high-yuanfen (*n* = 83) or low-yuanfen (*n* = 83) condition.

An independent-samples t-test showed that the perceived yuanfen level in the high-yuanfen condition (*M*_high_ = 6.40, *SD* = 0.60) was significantly higher than that in the low-yuanfen condition (*M*_low_ = 2.94, *SD* = 1.25), *t*(164) = −22.63, *p* < 0.001, confirming the success of the manipulation.

Further analysis revealed that participants in the high-yuanfen condition also reported stronger voice intention (α = 0.91; *M*_high_ = 5.85, *SD* = 0.58) than those in the low-yuanfen condition (*M*_low_ = 4.80, *SD* = 1.36), *t*(164) = −6.48, *p* < 0.001.

An ANCOVA controlling for gender, age, occupation, education, monthly income, and P2P stay frequency revealed that the main effect of yuanfen perception remained significant, *F*(1, 158) = 39.16, *p* < 0.001, η^2^ = 0.20. None of the control variables showed significant effects. These findings indicate that higher yuanfen perception significantly enhanced customers’ voice intention, supporting Hypothesis 1.

To test Hypothesis 2, the mediating role of social bond in the relationship between yuanfen perception and customers’ voice intention was examined using PROCESS Model 4. Gender, age, occupation, education, monthly income, and frequency of P2P accommodation stays were controlled. A bias-corrected bootstrap with 5,000 resamples and a 95% confidence interval (CI) was used.

The total effect of yuanfen perception on voice intention was significant (Effect = 1.03, *SE* = 0.16, *t* = 6.26, *p* < 0.001). After including social bond, the direct effect became nonsignificant (Effect = −0.36, *SE* = 0.22, *t* = −1.65, *p* = 0.10). However, the indirect effect through social bond was significant (Effect = 1.38, BootSE = 0.21,), with the CI excluding zero. These results indicate that social bond significantly mediates the effect of yuanfen perception on customers’ voice intention. The results remained consistent when control variables were excluded, thereby supporting Hypothesis 2 (see Table 2 for details).

**Table 2:** The analysis of the mediating effect of social bond (study 2a).

Independent variables	Social bond (mediator)			Customer voice intention (dependent variable)		
	Coefficient	SE	*p*	Coefficient	SE	*p*
Constant	0.812	0.849	0.341	4.424	0.689	0.000
Perceived yuanfen (PY)	2.595	0.170	0.000	−0.357	0.216	0.101
Social bond				0.533	0.064	0.000
Control variable: stay experience	0.027	0.092	0.768	0.074	0.074	0.320
Control variable: gender	0.261	0.179	0.147	−0.049	0.146	0.739
Control variable: age	−0.097	0.128	0.447	−0.051	0.104	0.625
Control variable: occupation	−0.004	0.088	0.968	−0.247	0.187	0.235
Control variable: education	−0.024	0.194	0.902	−0.180	0.155	0.247
Control variable: income	−0.026	0.087	0.769	−0.071	0.070	0.317
	R^2^ = 0.608; *F*(7,158) = 35.061, *p* = 0.000			*R^2^ =* 0.465; *F*(8, 157) = 17.045, *p* = 0.000		

Effect types	Effect value	SE	[LL, UL]
Total effect	1.027	0.164	[0.703, 1.351]
Direct effect	−0.357	0.216	[−0.784, 0.070]
Indirect effect (via social bond)	1.384	0.213	[0.998, 1.837]

SE, standard error; LL, lower limit of 95% confidence interval; UL, upper limit of 95% confidence.

### 4.2 Study 2b key card-based stimuli

#### 4.2.1 Experimental design and procedure

Building on the previous experiment, this study used key card-based stimuli instead of text to further explore how yuanfen perception influences customers’ voice intention. A one-factor between-subjects design was employed (yuanfen perception: high vs. low), with social bond as the mediating variable. A total of 200 participants were recruited.

Participants first read a description of the P2P accommodation’s functional quality. Following (Tang and Fong, 2024) the scenario stated: “After the first night’s stay, you find that the room has excellent soundproofing. The accommodation provides modern bathroom facilities with a well-functioning shower. The internet speed is fast but unstable.” Participants were then randomly assigned to either the high- or low-yuanfen condition.

Functional quality perception was measured using three items on a seven-point semantic differential scale (Ren et al., 2018): “poor/excellent,” “inferior/superior,” and “low standards/high standards.” The manipulation check followed the same procedure as in previous studies. Participants then completed measures of perceived functional quality, social bond, voice intention, and demographic information (gender, age, monthly income, education level, and occupation). The frequency of P2P accommodation stays during the past year was included as a control variable. All scales were consistent with those used in Study 2a.

#### 4.2.2 Results

A total of 200 questionnaires were collected. After excluding invalid responses, A total of 197 valid responses were retained (68.5% female, 31.5% male). Participants were randomly assigned to the high-yuanfen (*n* = 99) or low-yuanfen (*n* = 98) condition.

An independent-samples t-test showed that perceived yuanfen was significantly higher in the high-yuanfen condition (*M*_high_ = 5.66, *SD* = 0.75) than in the low-yuanfen condition (*M*_low_ = 4.78, *SD* = 1.13), *t*(195) = −6.48, *p* < 0.001, confirming that the manipulation was successful.

The difference in perceived functional quality (*α* = 0.86) between the high-yuanfen (*M*_high_ = 5.55, *SD* = 0.67) and low-yuanfen (*M*_low_ = 5.41, *SD* = 0.78) conditions was not significant, *t*(195) = −1.32, *p* = 0.187. This indicates that participants’ evaluations of functional quality did not differ significantly across conditions, ruling out potential confounding effects at the functional level.

Further analysis examined the link between yuanfen perception and customers’ voice intention (α = 0.86). Participants in the high-yuanfen condition reported significantly stronger voice intention (*M*_high_ = 5.77, *SD* = 0.63) than those in the low-yuanfen condition (*M*_low_ = 5.35, *SD* = 1.06), *t*(195) = −3.31, *p* = 0.001. These results suggest that high-yuanfen images not only increased perceived yuanfen but also strengthened participants’ willingness to express voice intention.

After controlling for demographics, stay frequency, and perceived functional quality, the effect of yuanfen perception remained significant, *F*(1, 190) = 9.14, *p* = 0.003, η^2^ = 0.046. Perceived functional quality also had a significant effect, *F*(1, 190) = 12.85, *p* < 0.001, η^2^ = 0.063, while stay frequency showed a marginal effect, *F*(1, 190) = 3.72, *p* = 0.055, η^2^ = 0.019. Even after controlling for demographic variables and perceived functional quality, yuanfen perception remained a significant positive predictor of voice intention, confirming the robustness of the findings.

To test the mediating role of social bond (*α* = 0.85) in the relationship between yuanfen perception and customers’ voice intention, PROCESS Model 4 was employed. A bias-corrected bootstrap with 5,000 resamples and a 95% confidence interval (CI) was used, controlling for gender, age, occupation, education, monthly income, frequency of P2P accommodation stays, and perceived functional quality.

The total effect of yuanfen manipulation on voice intention was significant (Effect = 0.362, *SE* = 0.121, *t* = 2.998, *p* = 0.003). After including social bond, the direct effect became nonsignificant (Effect = 0.162, *SE* = 0.102, *t* = 1.595, *p* = 0.113). However, the indirect effect through social bond was significant (Effect = 0.200, BootSE = 0.087,), and the standardized indirect effect was 0.224 (BootSE = 0.087). Hypothesis 2 was supported (see Table 3 for details).

**Table 3:** The analysis of the mediating effect of social bond (study 2b).

Independent variables	Social bond (mediator)		Customer voice intention (dependent variable)			
	Coefficient	SE	*p*	Coefficient	SE	*p*
Constant	1.506	0.921	0.104	2.488	0.682	0.000
Perceived yuanfen (PY)	0.392	0.135	0.004	0.162	0.102	0.113
Social bond				0.509	0.054	0.000
Control variable: Stay experience	0.148	0.072	0.041	0.049	0.053	0.356
Control variable: gender	−0.140	0.151	0.357	−0.035	0.112	0.755
Control variable: age	0.100	0.082	0.226	0.081	0.061	0.182
Control variable: occupation	−0.052	0.074	0.481	0.022	0.054	0.689
Control variable: education	−0.024	0.156	0.650	−0.027	0.115	0.812
Control variable: income	0.101	0.063	0.111	−0.047	0.047	0.315
Control variable: function experience	0.513	0.094	0.000	0.029	0.074	0.698
	R^2^ = 0.263; *F*(8,188) = 8.382, p = 0.000		R^2^ = 0.431; *F*(9,187) = 15.738, p = 0.000			

Effect types	Effect value	SE	[LL, UL]
Total effect	0.362	0.121	[0.124, 0.600]
Direct effect	0.162	0.102	[−0.038, 0.362]
Indirect effect (via social bond)	0.200	0.086	[0.059, 0.397]

SE, standard error; LL, lower limit of 95% confidence interval; UL, upper limit of 95% confidence.

## 5 Study 3

### 5.1 Study 3a text-based stimuli

#### 5.1.1 Experimental design and procedure

Study 3 examined the moderating role of perceived customer effectiveness. Participants were randomly assigned to either the high or low yuanfen condition. After reading the experimental materials (identical to those used in Study 2a), they sequentially completed the manipulation check, social bond, perceived customer effectiveness, and customers’ voice intention measures. The manipulation check and control variables were identical to those in Study 2a.

Perceived customer effectiveness was measured using a four-item scale adapted from Jaiswal and Kant (2018). A sample item was, “I feel that I am capable of helping the other party solve problems.” All items were rated on a seven-point Likert scale (1 = strongly disagree, 7 = strongly agree).

#### 5.1.2 Results

A total of 240 questionnaires were collected. Among the participants, 64.1% were female and 33.9% were male. All participants passed the attention check. Participants were randomly assigned to the high-yuanfen (*n* = 120) or low-yuanfen (*n* = 120) condition.

An independent-samples *t*-test revealed that perceived yuanfen was significantly higher in the high-yuanfen condition (*M*_high_ = 6.28, *SD* = 0.77) than in the low-yuanfen condition (*M*_low_ = 3.88, *SD* = 1.34), *t*(238) = −16.64, *p* < 0.001, confirming the success of the manipulation.

To test the hypothesis, PROCESS Model 14 was employed, controlling for perceived functional quality, gender, age, occupation, education, monthly income, and frequency of P2P accommodation stays.

The results revealed a significant interaction between social bond (*α* = 0.92) and perceived customer effectiveness (α = 0.77) on voice intention (α = 0.90), *b* = −0.107, *SE* = 0.034, *t* = −3.14, *p* = 0.002, *F*(10, 229) = 29.24, R^2^ = 0.561.

Simple slope analysis showed that when perceived customer effectiveness was low, the positive effect of social bond on voice intention was strongest (*b* = 0.481, *SE* = 0.056, *p* < 0.001). The effect remained significant at the medium level (*b* = 0.389, *SE* = 0.046, *p* < 0.001) but weakened at the high level (*b* = 0.297, *SE* = 0.053, *p* < 0.001).

**Figure 2:**
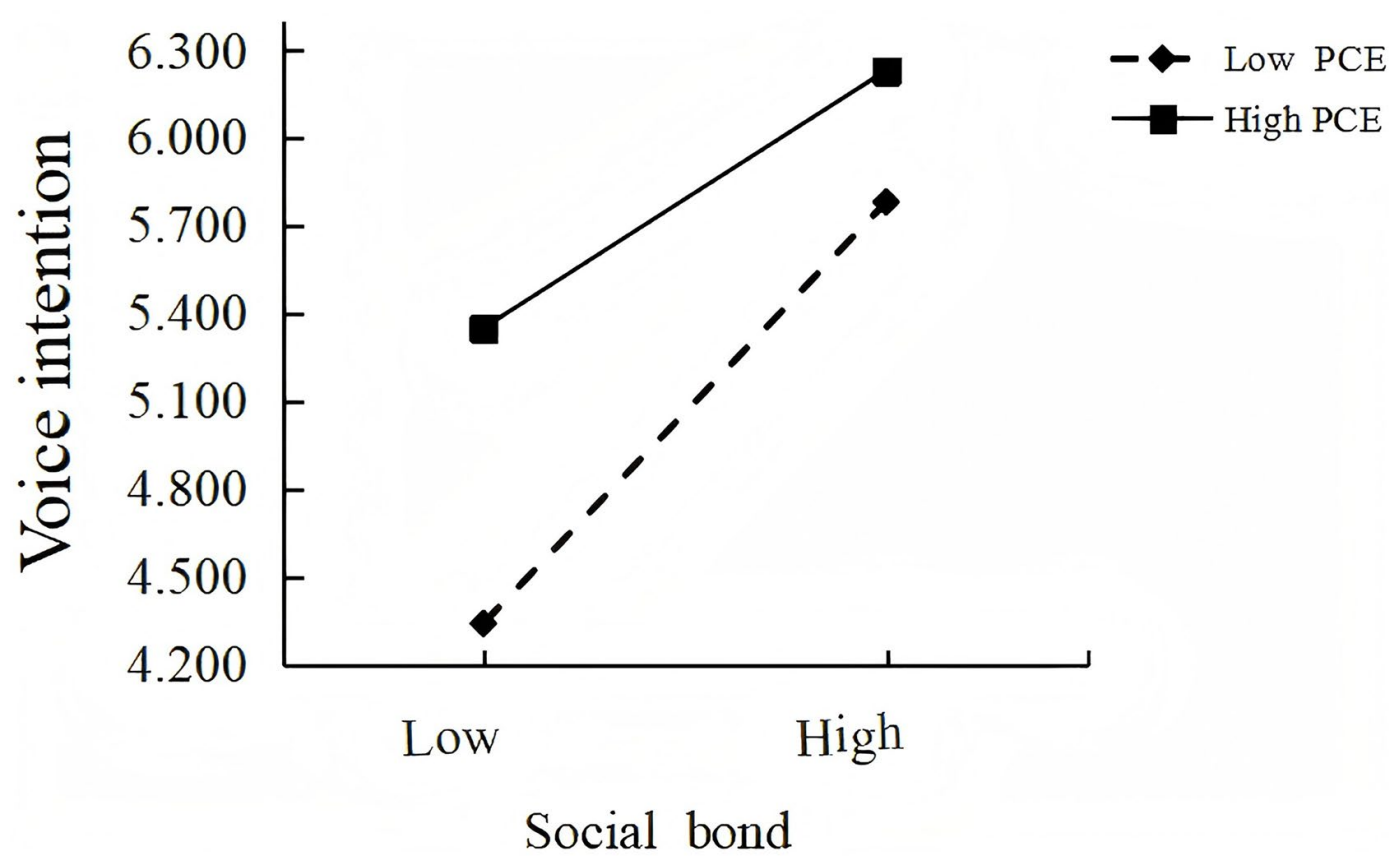
The moderating effect of PCE (study 3a).

The moderated mediation index was significant (Index = −0.209, BootSE = 0.078), suggesting that perceived customer effectiveness significantly moderated the mediating role of social bond. The indirect effect of yuanfen manipulation on voice intention via social bond was stronger under low perceived customer effectiveness. Therefore, Hypothesis 3 was not supported (Figure 2).

### 5.2 Study 3b key card-based stimuli

#### 5.2.1 Experimental design and procedure

Study 3 examined the moderating role of perceived customer effectiveness. The experiment employed a one-factor between-subjects design (yuanfen perception: high vs. low), with 240 participants randomly assigned to either the high- or low-yuanfen condition. After reading the scenario materials (identical to those in Study 2b), participants completed the manipulation check, followed by measures of the social bond, perceived customer effectiveness, and customers’ voice intention.

Functional quality perception was assessed using 37-point semantic differential items (Ren et al., 2018): “poor/excellent,” “inferior/superior,” and “low standards/high standards.” The measurement of other variables was identical to that used in Study 3a. All items were rated on a seven-point Likert scale (1 = strongly disagree, 7 = strongly agree).

#### 5.2.2 Results

A total of 240 questionnaires were distributed. After excluding responses that failed the attention check or were incomplete, 227 valid samples remained. Among the participants, 75 were male (33%) and 152 were female (67%). Participants were randomly assigned to the high-yuanfen (*n* = 116) or low-yuanfen (*n* = 111) condition.

An independent-samples t-test showed that participants in the high-yuanfen condition reported significantly higher perceived yuanfen (*M*_high_ = 5.74, *SD* = 0.87) than those in the low-yuanfen condition (*M*_low_ = 4.92, *SD* = 0.96), *t*(225) = −6.80, *p* < 0.001, confirming the success of the manipulation.

The difference in perceived functional quality (α = 0.87) between the high-yuanfen (*M*_high_ = 5.42, *SD* = 0.89) and low-yuanfen (*M*_low_ = 5.39, *SD* = 0.89) conditions was not significant, *t*(225) = −0.30, *p* = 0.767.

To test the proposed moderated mediation model, PROCESS Model 14 was employed, controlling for perceived functional quality, gender, age, occupation, education, monthly income, and frequency of P2P accommodation stays. The analysis revealed a significant interaction between social bond (α = 0.84) and perceived customer effectiveness (α = 0.75) on voice intention (α = 0.80), *F*(1, 215) = 24.60, *p* < 0.001, η^2^ = 0.09.

A simple-slope analysis further revealed that when perceived customer effectiveness was low, social bond had a significantly positive effect on voice intention (*b* = 0.140, *SE* = 0.049). The effect remained significant at the medium level of perceived customer effectiveness (*b* = 0.084, *SE* = 0.034) but became nonsignificant at the high level (*b* = 0.027, SE = 0.029, 95% CI [−0.027, 0.088]).

**Figure 3:**
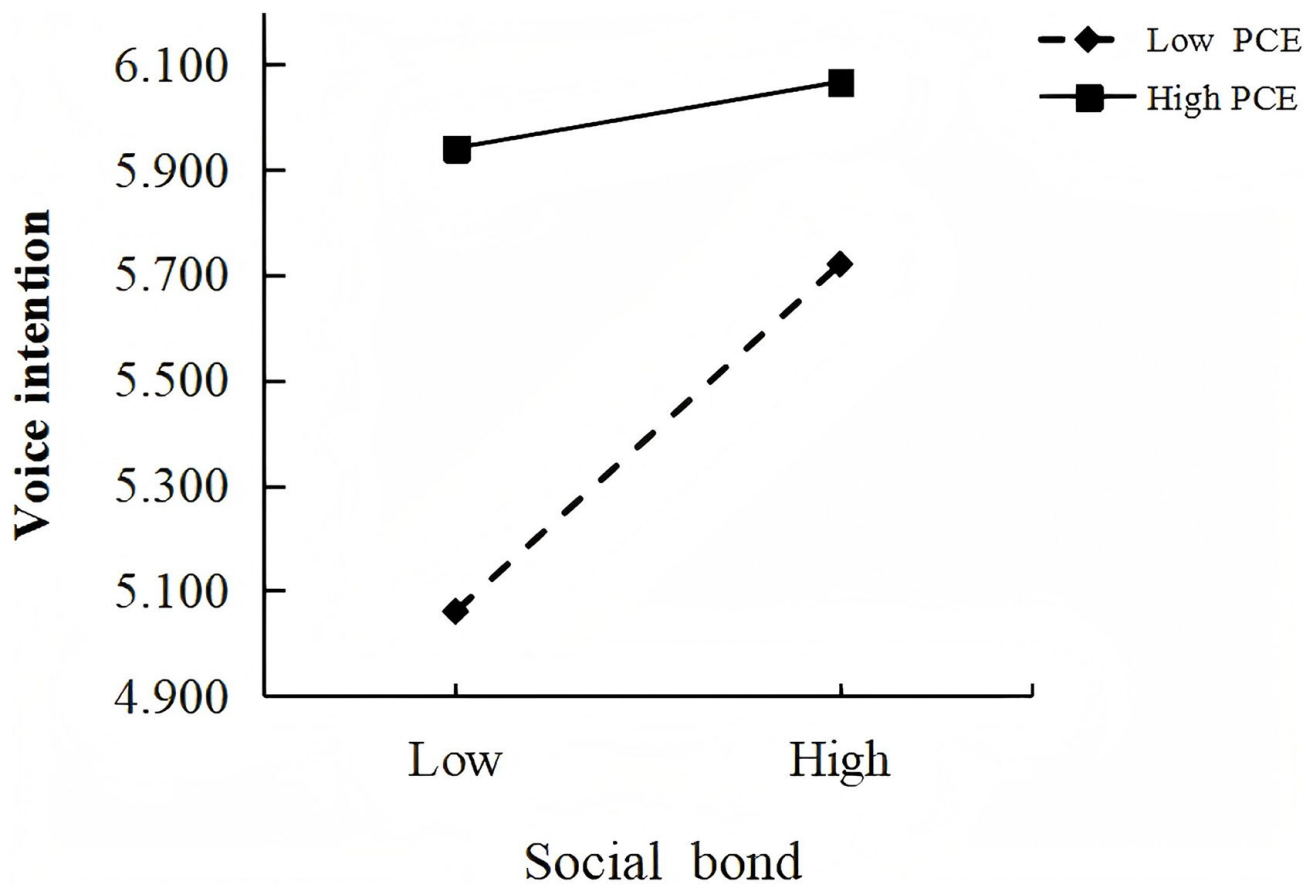
The moderating effect of PCE (study 3b).

The index of moderated mediation was significant (Index = −0.071, BootSE = 0.026), indicating that perceived customer effectiveness significantly moderated the mediating effect of social bond on customers’ voice intention. Therefore, Hypothesis 3 was not supported (Figure 3).

## 6 Conclusion and discussions

### 6.1 Conclusion

Grounded in CAT, this study examines how perceived yuanfen shapes customers’ voice intention through social bonds and perceived customer effectiveness. Experimental results confirm the main, mediating, and moderating effects among these variables, revealing how cognitive and emotional factors jointly drive positive customer behavior.

First, the main effect shows that perceived yuanfen significantly increases customers’ intention to provide feedback. Whether triggered by interpersonal similarities or symbolic object cues (e.g., room cards), a heightened sense of yuanfen motivates customers to engage in voice behavior. This aligns with CAT, demonstrating that cultural-cognitive appraisals of “destined connection” can evoke positive behavioral responses (Tang and Fong, 2024).

Second, the mediation analysis shows that social bonds play a crucial mediating role between perceived yuanfen and voice intention. When customers perceive strong yuanfen in P2P, this perception fosters stronger social bonds, which in turn enhance their willingness to provide constructive suggestions. In other words, social bonds represent the key psychological mechanism through which perceived yuanfen translates into positive voice intention. This finding extends research on affective mechanisms in customer–provider relationships, highlighting how emotional bonds encourage customers to express their opinions.

Furthermore, regarding the moderating role of PCE, our findings reveal an intriguing “functional substitution” effect, contrary to the initially hypothesized enhancing effect. While we expected high efficacy to amplify social bonds, the results consistently show that social bonds exert the strongest influence on voice intention when customers perceive low effectiveness. In other words, when customers doubt their cognitive capacity to make a difference (low PCE), the emotional connection derived from yuanfen acts as a compensatory motivational resource, driving them to speak up. Conversely, when PCE is high, the strong internal cognitive drive renders the additional impact of social bonds less critical. This highlights a complex, compensatory interplay where cultural-emotional bonds serve as a vital safety net for engagement precisely when functional confidence is lacking.

### 6.2 Theoretical contributions

Theoretical implications lie in three directions. First, this study advances theorizing on customer voice by reframing it through the lens of regenerative co-creation. Whereas prior research often treats customer voice as a generic form of citizenship behavior (Dong and Sivakumar, 2017), our findings suggest that yuanfen-driven voice operates as a relationally regenerative micro-mechanism. Tourism serves not only as a context for transient interactions but also as a space for building social capital through benevolent acts (Glover and Filep, 2015). Given that P2P hosts are vital change agents in community development (Bellato et al., 2022), such voice-based support carries implications beyond conventional customer citizenship behavior. Specifically, voice may serve a function akin to relational restoration by helping repair, renew, and strengthen host–guest ties (Vegas-Macias et al., 2026). This distinguishes voice from purely transactional feedback; instead, it reflects co-creative engagement that can nurture the vitality of hosts’ micro-businesses through both instrumental and socio-emotional pathways. Collaborative participation lies at the core of regenerative processes, with stakeholders in regenerative tourism collaborating by sharing roles, responsibilities, knowledge, tasks, and resources (Bellato et al., 2023). This perspective aligns with regenerative tourism scholarship that calls for greater attention to its socio-cultural dimensions (Zhang et al., 2023a; Vegas-Macias et al., 2026). Our results elevate customer voice from “helpful feedback” to renewal-oriented participation that promotes the well-being and harmony of local communities. Moreover, when hosts respond positively to voice, customers often feel valued and motivated. This can lead to revisit intentions, positive word-of-mouth, or continued voice behavior in other destinations. All of these outcomes illustrate tourism as a “force for good.”

Moreover, building on cultural perspectives, this study introduces yuanfen as a new novel lens for interpreting customer voice in P2P accommodation. Responding to calls for exploring yuanfen perceptions in physical spaces (Tang et al., 2024), we empirically demonstrate how this construct operates through both interpersonal and object-based cues to foster social bonds. Crucially, we differentiate yuanfen from adjacent constructs such as serendipity, karma, and destiny beliefs. Serendipity refers to interpretations of unexpected events and positive surprise experiences (Kim et al., 2021); karma represents a moralized causal belief system that attributes outcomes to ethical actions (Xue and Mattila, 2024); and destiny beliefs capture an implicit theory that relationships are either “meant to be” or not (Rai et al., 2017). However, yuanfen represents an attributional appraisal that interprets the initiation and development of relationships as predestined, distinct from karma’s emphasis on causal cycles and moral retribution. Within the yuanfen framework, there is no explicit notion of “cause”; rather, an inexplicable force brings individuals together. Unlike destiny beliefs,yuanfen represents an active attributional mindset: it encourages individuals not only to accept the connection but to actively invest in nurturing it (Tang et al., 2024). This study thus clarifies how yuanfen serves as a culturally specific antecedent that transforms a chance encounter into a meaningful social bond, offering a robust foundation for future research on fate-related relational dynamics in tourism.

Furthermore, the counterintuitive finding regarding Perceived Customer Effectiveness (PCE) offers a nuanced refinement to Cognitive Appraisal Theory. Traditional applications of CAT often imply a synergistic logic in which higher coping potential strengthens the translation of emotional appraisals into behavioral intentions (Smith and Lazarus, 1993). our results reveal a functional substitution mechanism. Specifically, we find that social bonds exert their strongest influence on voice intention when customers perceive low effectiveness in P2P accommodations. This suggests that cognitive resources perceived customer effectiveness and relational resources (social bonds) may operate in a compensatory manner (Hobfoll, 1989). When perceived effectiveness is high, customers are more likely to rely on an agentic logic in which internal confidence drives behavioral engagement. Under such conditions, the additional motivational contribution of relational bonds becomes less critical. In contrast, when perceived effectiveness is low, customers lack confidence in their instrumental impact; relational bonds then become a salient motivational resource that compensates for this perceived limitation and encourages voice. Importantly, this finding does not contradict CAT but refines it by identifying boundary conditions under which primary affective appraisals and secondary coping appraisals interact. Rather than always amplifying emotional pathways, secondary appraisals may sometimes reduce the relative necessity of relational motivation. By revealing this compensatory dynamic, our study extends prior tourism research that predominantly assumes synergistic effects (e.g., Choi and Choi, 2019) and clarifies when cultural relational mechanisms are most behaviorally consequential.

### 6.3 Practical implications

This study offers practical implications in three key areas. First, by identifying the role of perceived yuanfen, it provides cultural informed insights into Chinese customers. The findings show that whether experienced through interpersonal or physical-environmental cues, perceived yuanfen is often interpreted as a sense of meaningful connectedness, which can motivate customers to share constructive feedback and recommendations. Practitioners can leverage this by acknowledging situational coincidences—such as common interests, experiences, or hometowns—and framing them in terms of yuanfen to foster emotional connection. Moreover, physical design elements may also evoke yuanfen-based perceptions; incorporating subtle visual or textual cues related to yuanfen in room cards or décor may help activate culturally resonant interpretations and enhance customers’ emotional engagement.

Second, the study finds that yuanfen strengthens social bonds, which subsequently enhance customers’ voice intention. In a preliminary exploration of yuanfen perceptions in P2P accommodations, guests often described casual conversations and warm hospitality as creating memorable yuanfen experiences. Because yuanfen is frequently associated with positive emotional resonance, service enthusiasm and lifestyle-oriented communication may be effective ways to evoke it. Strengthened social bonds can further encourage customers to share suggestions or recommendations. Practitioners may foster such bonds by maintaining appropriate post-stay relational touchpoints—for example, following up with guests, sharing helpful local travel tips, or creating a warm and home-like atmosphere that sustains a sense of connection.

Third, the study shows that customers’ perceived customer effectiveness moderates the relationship between social bonds and customers’ voice intention. In practice, managers may adopt differentiated strategies based on customers’ levels of perceived customer effectiveness. For customers with lower perceived effectiveness, emotional interaction and personalized communication may help strengthen social bonds and increase confidence in sharing feedback. In contrast, for customers with higher perceived effectiveness, emotional engagement may be less critical. Managers may instead provide more structured feedback mechanisms, such as brief surveys or clearly designed review prompts, to facilitate rational and constructive customer voice.

### 6.4 Limitations and future research

Although this study advances understanding of yuanfen perception in P2P accommodation, several limitations suggest directions for future research. First, the scenario-based experimental design, while enhancing internal validity and manipulation precision, raises concerns about ecological validity, interaction authenticity, and the nature of yuanfen activation. The present findings are limited to customers’ voice intentions rather than enacted voice behaviors, and it remains unclear whether the experimental stimuli activated pre-existing cultural beliefs about yuanfen or temporarily constructed such perceptions. Moreover, real host–guest and guest–object encounters in P2P accommodation are dynamic, reciprocal, and unfold over time, often involving repeated stays, ongoing online communication, direct messaging, and post-stay reviews (Farmaki et al., 2020). These elements may amplify, attenuate, or qualitatively alter the effects of yuanfen on actual voice behavior in ways not captured by hypothetical scenarios. Future research should collaborate with P2P platforms and employ longitudinal or field designs to examine whether yuanfen cues predict enacted customer voice (e.g., private messages, public reviews, or suggestions). Second, the cultural boundary conditions of yuanfen require clearer specification. Because our studies were conducted in China with Chinese-language materials and a Chinese sample, generalizability to non-Chinese contexts remains limited. Future research should extend the cultural boundary conditions by recruiting diverse and bicultural samples with measures of acculturation. Such designs would clarify whether the appraisal–emotion–voice pathway is culturally specific, or if functionally analogous constructs operate differently across cultural contexts. Third, our person–object operationalization relied on a specific artifact (a key card). As such objects vary in symbolic meaning across spaces, future studies should test whether yuanfen perceptions differ across public vs. private areas and communal vs. personal objects to clarify when physical environments and material cues evoke culturally grounded relational appraisals.

## Data Availability

The raw data supporting the conclusions of this article will be made available by the authors, without undue reservation.

## References

[ref1] Aron E. N. Aron A. (1996). Love and expansion of the self: the state of the model. Pers. Relat. 3, 45–58. doi: 10.1111/j.1475-6811.1996.tb00103.x

[ref2] Assaf A. G. Josiassen A. Knežević Cvelbar L. Woo L. (2015). The effects of customer voice on hotel performance. Int. J. Hosp. Manag. 44, 77–83. doi: 10.1016/j.ijhm.2014.09.009

[ref3] Assaf A. G. Kock F. Tsionas M. (2022). Tourism during and after COVID-19: an expert-informed agenda for future research. J. Travel Res. 61, 454–457. doi: 10.1177/00472875211017237

[ref4] Bandura S. (1977). *Self-efficacy: Toward a Unifying theory of Behavioral Change*. Available at: https://psycnet.apa.org/record/1977-25733-001 (Accessed February 9, 2026). 10.1037//0033-295x.84.2.191 847061

[ref5] Béal M. Sabadie W. (2018). The impact of customer inclusion in firm governance on customers’ commitment and voice behaviors. J. Bus. Res. 92, 1–8. doi: 10.1016/j.jbusres.2018.07.019

[ref6] Bellato L. Frantzeskaki N. Briceño Fiebig C. Pollock A. Dens E. Reed B. (2022). Transformative roles in tourism: adopting living systems’ thinking for regenerative futures. JTF 8, 312–329. doi: 10.1108/JTF-11-2021-0256

[ref7] Bellato L. Frantzeskaki N. Nygaard C. A. (2023). Regenerative tourism: a conceptual framework leveraging theory and practice. Tour. Geogr. 25, 1026–1046. doi: 10.1080/14616688.2022.2044376

[ref8] Bellocchi A. (2022). Science students’ social bonds and knowledge construction. J. Res. Sci. Teach. 59, 746–778. doi: 10.1002/tea.21743

[ref9] Bove L. L. Pervan S. J. Beatty S. E. Shiu E. (2009). Service worker role in encouraging customer organizational citizenship behaviors. J. Bus. Res. 62, 698–705. doi: 10.1016/j.jbusres.2008.07.003

[ref10] Bove L. L. Robertson N. L. (2005). Exploring the role of relationship variables in predicting customer voice to a service worker. J. Retail. Consum. Serv. 12, 83–97. doi: 10.1016/j.jretconser.2004.03.003

[ref11] Carr C. T. Rosaen S. F. (2024). We’re going streaking!: associations between the gamification of mediated communication and relational closeness. Commun. Rep. 37, 185–199. doi: 10.1080/08934215.2024.2334743

[ref12] Chang H.-C. Holt G. R. (1991). “The concept of yuan and Chinese interpersonal relationships,” in Cross-cultural interpersonal communication, eds. Ting-Toomey S. Korzenny F. (Thousand Oaks, CA: Sage Publications, Inc), 28–57.

[ref13] Chen S. Chen W. Luo X. (2023). Some stay and some quit: understanding P2P accommodation providers’ continuous sharing behavior from the perspective of feedback theory. Tour. Manag. 95:104676. doi: 10.1016/j.tourman.2022.104676

[ref14] Chen G. Li S. (2021). Effect of employee–customer interaction quality on customers’ prohibitive voice behaviors: mediating roles of customer trust and identification. Front. Psychol. 12:773354. doi: 10.3389/fpsyg.2021.773354, 34970197 PMC8712316

[ref15] Choi H. Choi H. C. (2019). Investigating tourists’ fun-eliciting process toward tourism destination sites: an application of cognitive appraisal theory. J. Travel Res. 58, 732–744. doi: 10.1177/0047287518776805

[ref16] deMatos N. M. S. Sá E. S. Duarte P. A. (2021). A review and extension of the flow experience concept. Insights and directions for tourism research. Tour. Manag. Perspect. 38:100802. doi: 10.1016/j.tmp.2021.100802

[ref17] Dong B. Sivakumar K. (2017). Customer participation in services: domain, scope, and boundaries. J. of the Acad. Mark. Sci. 45, 944–965. doi: 10.1007/s11747-017-0524-y

[ref18] Efe F. O. Tumer E. Aksoy N. C. Alan A. K. (2025a). Customer voice behavior: a systematic review and future research agenda. Int. J. Consum. Stud. 49:e70142. doi: 10.1111/ijcs.70142

[ref19] Efe F. O. Tumer E. Aksoy N. C. Alan A. K. (2025b). Guest voice behavior for hotels: the role of experience, emotional attachment, and perceived value. Cornell Hosp. Q. 67:401. doi: 10.1177/19389655251367401

[ref20] Eisingerich A. B. Auh S. Merlo O. (2014). Acta non verba? The role of customer participation and word of mouth in the relationship between service firms’ customer satisfaction and sales performance. J. Serv. Res. 17, 40–53. doi: 10.1177/1094670513490836

[ref21] Ellen P. S. Wiener J. L. Cobb-Walgren C. (1991). The role of perceived consumer effectiveness in motivating environmentally conscious behaviors. J. Public Policy Mark. 10, 102–117. doi: 10.1177/074391569101000206

[ref22] Fang S. Han X. Chen S. (2023). The impact of tourist–robot interaction on tourist engagement in the hospitality industry: a mixed-method study. Cornell Hosp. Q. 64, 246–266. doi: 10.1177/19389655221102383

[ref23] Farmaki A. Christou P. Saveriades A. (2020). A Lefebvrian analysis of Airbnb space. Ann. Tour. Res. 80:102806. doi: 10.1016/j.annals.2019.102806

[ref24] Glover T. D. Filep S. (2015). On kindness of strangers in tourism. Ann. Tour. Res. 50, 159–162. doi: 10.1016/j.annals.2014.10.001

[ref25] Gong T. Choi J. N. (2016). Effects of task complexity on creative customer behavior. EJM 50, 1003–1023. doi: 10.1108/EJM-04-2015-0205

[ref26] Heider F. (1958). The Psychology of Interpersonal Relations. New York: John Wiley & Sons. doi: 10.1037/10628-000

[ref27] Hibbard J. D. Kumar N. Stern L. W. (2001). Examining the impact of destructive acts in Marketing Channel relationships. J. Mark. Res. 38, 45–61. doi: 10.1509/jmkr.38.1.45.18831

[ref28] Hirschman A. O. (1970). Exit, Voice, and Loyalty: Responses to Decline in Firms, Organizations, and states. Cambridge, MA: Harvard University Press.

[ref29] Hobfoll S. E. (1989). Conservation of Resources. A new attempt at conceptualizing stress. American Psychologist, 44, 513–524. doi: 10.1037/0003-066X.44.3.513 2648906

[ref30] Hsu H.-P. Hwang K.-K. (2016). Serendipity in relationship: a tentative theory of the cognitive process of Yuanfen and its psychological constructs in Chinese cultural societies. Front. Psychol. 7:82. doi: 10.3389/fpsyg.2016.00282, 26973576 PMC4771764

[ref31] Iddawala J. Lee D. (2025). Regenerative tourism: context and Conceptualisations. Tour. Plan. Dev. 2024, 1–31. doi: 10.1080/21568316.2025.2527614

[ref32] Jaiswal D. Kant R. (2018). Green purchasing behaviour: a conceptual framework and empirical investigation of Indian consumers. J. Retail. Consum. Serv. 41, 60–69. doi: 10.1016/j.jretconser.2017.11.008

[ref33] Jiang Z. Chen R. (2025). To vote or not to vote? The impact of gratitude expression on helpfulness voting in peer-to-peer accommodation reviews. Tour. Manag. 108:105094. doi: 10.1016/j.tourman.2024.105094

[ref34] Jiang T. Miao L. Fu X. (2022). Tourism and yuan-based strangership. Ann. Tour. Res. 94:103401. doi: 10.1016/j.annals.2022.103401

[ref35] Jonsson P. Zineldin M. (2003). Achieving high satisfaction in supplier-dealer working relationships. Supply Chain Manag. Int. J. 8, 224–240. doi: 10.1108/13598540310484627

[ref36] Kim A. Affonso F. M. Laran J. Durante K. M. (2021). Serendipity: chance encounters in the marketplace enhance consumer satisfaction. J. Mark. 85, 141–157. doi: 10.1177/00222429211000344

[ref37] Kumar N. Garg P. Singh S. (2022). Pro-environmental purchase intention towards eco-friendly apparel: augmenting the theory of planned behavior with perceived consumer effectiveness and environmental concern. J. Glob. Fash. Mark. 13, 134–150. doi: 10.1080/20932685.2021.2016062

[ref38] Lacey R. (2012). How customer voice contributes to stronger service provider relationships. J. Serv. Mark. 26, 137–144. doi: 10.1108/08876041211215293

[ref39] Lavuri R. (2022). Organic green purchasing: moderation of environmental protection emotion and price sensitivity. J. Clean. Prod. 368:133113. doi: 10.1016/j.jclepro.2022.133113

[ref40] Lee C. Hallak R. Sardeshmukh S. R. (2019). Creativity and innovation in the restaurant sector: supply-side processes and barriers to implementation. Tour. Manag. Perspect. 31, 54–62. doi: 10.1016/j.tmp.2019.03.011

[ref41] LePine J. A. Van Dyne L. (1998). Predicting voice behavior in work groups. J. Appl. Psychol. 83:853. doi: 10.1037/0021-9010.83.6.853

[ref42] Li F. S. Su Q. Guan J. Zhang G. (2023). Communicate like humans? Anthropomorphism and hotel consumers’ willingness to pay a premium price. J. Hosp. Tour. Manag. 56, 482–492. doi: 10.1016/j.jhtm.2023.08.008

[ref43] Liao G. Wang J. Zhang Q. Ding X. (2024). The quality of crowdsourcing virtual community and users’ voice behavior: an analysis of stimulus-organism-response framework among Chinese users. Heliyon 10:e26881. doi: 10.1016/j.heliyon.2024.e26881, 38434368 PMC10904280

[ref45] Liu X. Hu Y. Chen X. Zhang M. (2026). “Cyber lovers”: the impact of AI social chatbots on users’ emotional attachment. Curr. Psychol. 45:293. doi: 10.1007/s12144-025-08584-3

[ref46] Liu Y. Li Y. Song K. Chu F. (2024). The two faces of artificial intelligence (AI): analyzing how AI usage shapes employee behaviors in the hospitality industry. Int. J. Hosp. Manag. 122:103875. doi: 10.1016/j.ijhm.2024.103875

[ref47] Liu-Lastres B. Bao H. Cecil A. (2025). Exploring gaps in young generations’ sustainable travel behavior: a mixed-methods study. J. Sustain. Tour. 33, 1165–1180. doi: 10.1080/09669582.2025.2453693

[ref48] Meng B. Luo D. (2023). Family tourists’ emotional responses at world heritage sites (WHSs): the effects of existential authenticity and interpersonal interaction. Tour. Rev. 79:53. doi: 10.1108/TR-01-2023-0053

[ref49] Nasr L. Burton J. Gruber T. Kitshoff J. (2014). Exploring the impact of customer feedback on the well-being of service entities. J. Serv. Manag. 25, 531–555. doi: 10.1108/JOSM-01-2014-0022

[ref50] Nguyen D. M. Chiu Y.-T. H. (2023). Corporate social responsibility authenticity as an antecedent to customer citizenship behavior: evidence from hospitality industry in Taiwan. J. Hosp. Market. Manag. 32, 477–504. doi: 10.1080/19368623.2023.2188510

[ref51] Nguyen D. M. Yang A. J.-F. (2025). Cultivating consumer extra-role behaviors via green psychological benefits: the roles of gratitude and perceived consumer effectiveness. J. Hosp. Market. Manag. 34, 1017–1042. doi: 10.1080/19368623.2025.2509673

[ref52] Norenzayan A. Lee A. (2010). It was meant to happen: explaining cultural variations in fate attributions. J. Pers. Soc. Psychol. 98, 702–720. doi: 10.1037/a0019141 20438219

[ref53] Oertzen A. S. Odekerken-Schröder G. Brax S. A. Mager B. (2018). Co-creating services—conceptual clarification, forms and outcomes. J. Serv. Manag. 29, 641–679. doi: 10.1108/josm-03-2017-0067

[ref54] Osakwe C. N. Ogunmokun O. A. Adeola O. Jibril A. B. (2024). Cultural values and voice as determinants of customers’ marketing research cooperation: a fuzzy set perspective. Int. J. Consum. Stud. 48:e13055. doi: 10.1111/ijcs.13055

[ref9001] Pang J. Fu X. Qi Y. (2024). The role of peer-to-peer interactions in sharing accommodation: From cocreation experience to peer customer engagement. Int J Hospital Manag 120:103764. doi: 10.1016/j.ijhm.2024.103764

[ref55] Pang Z. Sun J. (2025). How does the host–guest trust relationship evolve in tourism destinations? A Yangshuo case study. Tour. Trib. 25:36. doi: 10.19765/j.cnki.1002-5006.2024.00.036

[ref56] Park J. Ahn S. Lee S. (2025). Pre-owned, still precious: how emotional attachment shapes pre-owned luxury consumption. J. Retail. Consum. Serv. 87:104420. doi: 10.1016/j.jretconser.2025.104420

[ref57] Park C. W. Macinnis D. J. Priester J. Eisingerich A. B. Iacobucci D. (2010). Brand attachment and brand attitude strength: conceptual and empirical differentiation of two critical brand equity drivers. J. Mark. 74, 1–17. doi: 10.1509/jmkg.74.6.1

[ref58] Peng S. Li C. Tu X. Fan X. (2026). The power of language in promotion: exploring the impact of language style and the moderating role of address forms. J. Retail. Consum. Serv. 89:104609. doi: 10.1016/j.jretconser.2025.104609

[ref59] Pera R. Viglia G. Grazzini L. Dalli D. (2019). When empathy prevents negative reviewing behavior. Ann. Tour. Res. 75, 265–278. doi: 10.1016/j.annals.2019.01.005

[ref60] Rai D. Lin C.-W. Hong J. Kulick G. (2017). The influence of relationship beliefs on gift giving. Manag. Mark. 12, 697–709. doi: 10.1515/mmcks-2017-0040

[ref61] Ran Y. Zhou H. (2019). How does customer–company identification enhance customer voice behavior? A moderated mediation model. Sustainability 11:4311. doi: 10.3390/su11164311

[ref62] Ran Y. Zhou H. (2020). Customer–company identification as the enabler of customer voice behavior: how does it happen? Front. Psychol. 11:777. doi: 10.3389/fpsyg.2020.00777, 32390920 PMC7189116

[ref63] Ren X. Xia L. Du J. (2018). Delivering warmth by hand: customer responses to different formats of written communication. JSM 32, 223–234. doi: 10.1108/JSM-04-2017-0133

[ref64] Smith C. A. Ellsworth P. C. (1985). Patterns of cognitive appraisal in emotion. J. Pers. Soc. Psychol. 48, 813–838. doi: 10.1037/0022-3514.48.4.813, 3886875

[ref65] Smith C. A. Lazarus R. S. (1993). Appraisal components, core relational themes, and the emotions. Cogn. Emot. 7, 233–269. doi: 10.1080/02699939308409189

[ref67] Su L. Wang X. Huang S. (2025). Does travel sharing type promote creativity? The serial mediation effect of self-concept clarity and self-efficacy. Tour. Manag. 110:105194. doi: 10.1016/j.tourman.2025.105194 Sam

[ref68] Tang X. Fong L. H. N. (2024). Does perceived yuanfen impact Chinese customers’ hotel ratings? Int. J. Hosp. Manag. 122:103871. doi: 10.1016/j.ijhm.2024.103871

[ref69] Tang X. Fong L. H. N. So A. S.-I. (2024). Toward a framework for perceived yuanfen in the accommodation service encounter: a grounded theory study. IJCHM 36, 155–181. doi: 10.1108/IJCHM-07-2022-0896

[ref70] Tao C. B. Deng B. Sun J. (2024). Memorable interactive experiences between hosts and guests in B&Bs and their generation process: the host perspective. Tour. Manag. 102:104861. doi: 10.1016/j.tourman.2023.104861

[ref71] Vegas-Macias J. Jørgensen M. T. Sørensen F. (2026). Enterprise-mediated social value creation through tourist-resident interaction in regenerative tourism experiences: the case of CopenPay. J. Sustain. Tour. 2, 1–28. doi: 10.1080/09669582.2026.2614536

[ref72] Wan W. Li H. (2021). Power drives consumer voice behavior. JCMARS 4, 22–43. doi: 10.1108/JCMARS-09-2020-0039

[ref73] Wang J. Chi M. Huang R. Li W. (2025). Factors and mechanisms influencing the resilience of peer-to-peer accommodation hosts during the COVID-19 crisis: insights from a qualitative study. Int. J. Mob. Commun. 26, 214–238. doi: 10.1504/IJMC.2025.148004

[ref74] Wang S. Ma E. Shi M. Xu J. Xu S. (2025). Designing supportive spaces: social bonds in P2P accommodations. Int. J. Contemp. Hosp. Manag. 37, 3004–3021. doi: 10.1108/IJCHM-01-2025-0006

[ref75] Wang X. Xie J. (2021). Yuanfen and traveling neo-tribes: social interactions and group relations among Chinese road travelers. J. Tourism Res. 23, 332–345. doi: 10.1002/jtr.2410

[ref76] Weiner B. (2014). The attribution approach to emotion and motivation: history, hypotheses, home runs, headaches/heartaches. Emotion Rev. 6, 353–361. doi: 10.1177/1754073914534502

[ref77] Wilson D. T. (1995). An integrated model of buyer-seller relationships. J. Acad. Mark. Sci. 23, 335–345. doi: 10.1177/009207039502300414

[ref78] Wong T. Y. T. Peko G. Sundaram D. Piramuthu S. (2016). Mobile environments and innovation co-creation processes & ecosystems. Inf. Manag. 53, 336–344. doi: 10.1016/j.im.2015.09.005

[ref79] Xue J. Xunyue Mattila A. S. (2024) Instant karma: how the karmic-investment mindset affects customer engagement with corporate charitable giving requests J. Hosp. Tourism Res. 48 770–782 doi: 10.1177/10963480221137779

[ref44] Yang B. Yu H. Yu Y. Liu M. (2021). Community experience promotes customer voice: Co-creation value perspective. Mark. Intell. Plan. 39, 825–841. doi: 10.1108/MIP-01-2021-0030

[ref80] Yan H. Chai H. (2021). Consumers’ intentions towards green hotels in China: an empirical study based on extended norm activation model. Sustainability 13:2165. doi: 10.3390/su13042165

[ref81] Yang H. (1998). Multiple equilibrium behaviors and advanced traveler information systems with endogenous market penetration. Transp. Res. Part B Methodol. 32, 205–218. doi: 10.1016/S0191-2615(97)00025-8

[ref82] Yang K. S. Ho D. Y. (1988). The role of yuan in Chinese social life: a conceptual and empirical analysis. Asian Contribut. Psychol. 263:81.

[ref83] Yang R. Song H. (2024). The spillover effect of Chinese sportswear brands’ relationship quality: a perspective of Confucian yuanfen culture. IJSMS 25, 1105–1125. doi: 10.1108/IJSMS-02-2024-0049

[ref84] Yang C. Sun Y. Wang N. Shen X.-L. (2024). Disentangling the antecedents of rational versus emotional negative electronic word of mouth on a peer-to-peer accommodation platform. INTR 34, 563–585. doi: 10.1108/INTR-02-2022-0120

[ref85] Yau O. (1994). Consumer Behavior in China. New York: Routledge.

[ref86] Yi J. J. Chen C. M. (2015). A literature review of psychic distance from information flow, perception and yuanfen perspectives and prospects. Foreign Econ. Manag. 37, 85–96. doi: 10.16538/j.cnki.fem.2015.05.001

[ref87] Zhang T. Bufquin D. Lu C. (2019). A qualitative investigation of microentrepreneurship in the sharing economy. Int. J. Hosp. Manag. 79, 148–157. doi: 10.1016/j.ijhm.2019.01.010

[ref88] Zhang G. Higham J. Albrecht J. N. (2023a). Ecological restoration and visitor experiences: insights informed by environmental philosophy. J. Sustain. Tour. 31, 1252–1270. doi: 10.1080/09669582.2021.1922424

[ref89] Zhang G. Jian X. Chen J. Liu Y. (2023b). Riding the storm: understanding how peer-to-peer accommodation hosts manage role stress and the host-guest relationship. J. Hosp. Tour. Manag. 57, 179–189. doi: 10.1016/j.jhtm.2023.10.006

[ref90] Zhang G. Liu Y. Cheng M. Du M. (2025). While I’m not here, I can still welcome you home: how space–guest interactions shape P2P accommodation appeal. Int. J. Contemp. Hosp. Manag. 37:1921. doi: 10.1108/IJCHM-12-2024-1921

[ref91] Zhang G. Wang R. Cheng M. (2020). Peer-to-peer accommodation experience: a Chinese cultural perspective. Tour. Manag. Perspect. 33:100621. doi: 10.1016/j.tmp.2019.100621

