# Supplementary material for: Culture as a catalyst for regenerative co-creation: the role of yuanfen in driving customer voice through social bonds in P2P accommodation
Source: Front Psychol. 2026 Mar 10;17:1759683. doi: 10.3389/fpsyg.2026.1759683 (PMC13008953; doi:10.3389/fpsyg.2026.1759683)
Supplement: Supplementary file 1 [file Table_1.DOCX]

***Supplementary Material***

**Appendix A: details in Study**

**（1）Text-Based Stimuli**

Please imagine that you are working in a city thousands of kilometers away from your hometown. During a holiday, you and your friends decide to take a short vacation in a nearby tourist destination (S). You choose to stay at a local peer-to-peer (P2P) accommodation.

**High yuanfen**

When you arrive at the accommodation, you feel an instant sense of connection with the host, as if you were old friends who haven’t seen each other for a long time. As the conversation goes on, you are pleasantly surprised to find out that you and the host are from the same hometown. You chat about some familiar details of your hometown, which makes you feel a strong sense of warmth and closeness. When you mention a local dish that you really like, the host also says it’s one of their favorites, and you both share fond memories of your hometown together.﻿

**Low yuanfen**

When you arrive at the accommodation, your conversation with the host is limited to a few polite greetings, and you don’t feel anything special. During the small talk, you learn that the host came from another city to start a business. You never manage to find any common topics, and the conversation remains rather ordinary. When you mention a hometown dish that you really like, the host says they have never tried it and are not familiar with it.

1. **Key cards -Based Stimuli**

**High yuanfen**

| Original Image | Translated Image |
| --- | --- |
| 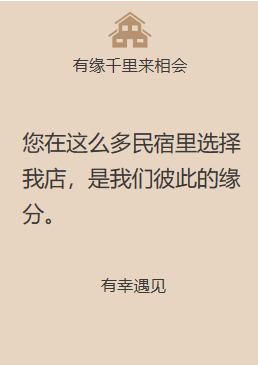 | **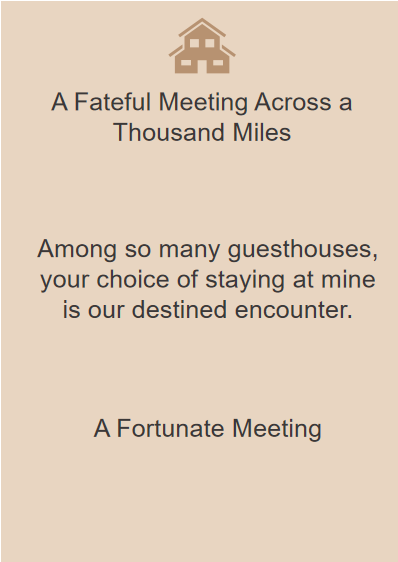** |

**Low yuanfen**

| Original Image | Translated Image |
| --- | --- |
| 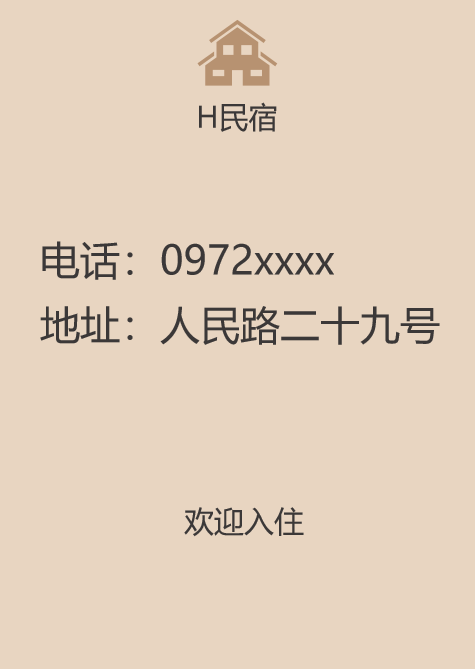 | **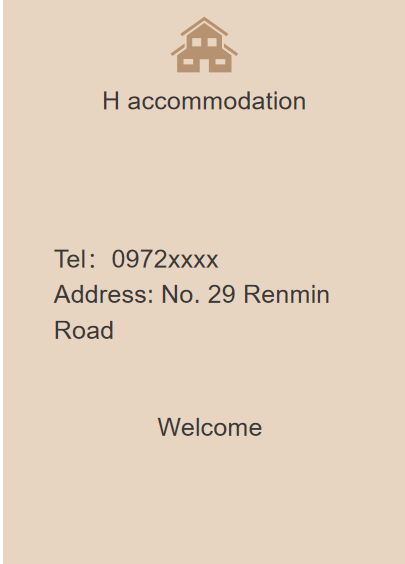** |

**(3) Measurement items**

| **Variables** | **Measurement Items** |
| --- | --- |
| Voice intention  (adapted from Bove et al., 2009) | I would make suggestions to my **host as to how the service could be improved at the accommodation.**  I would let my **host know of ways that they could better serve my needs during my stay.**  I would share my opinions with my **host if I felt they might be of benefit to the accommodation experience.**  I would contribute ideas to my **host that could improve service at the accommodation.** |
| Perceived yuanfen  (adapted from Tang & Fong, 2024) | I feel that I have yuanfen with this P2P accommodation/ host. |
| Social bond  (adapted from Fang et al., 2023) | I would like to interact directly with the **host**.  I would like to become friend with the **host**.  I would like to play with **host**. |
| Functional quality perception  (adapted from Ren et al., 2018) | This P2P accommodation is...  poor/excellent;  inferior/superior;  low standards/high standards. |
| Perceived customer effectiveness  (adapted from Jaiswal & Kant, 2018) | Each person's behavior can have a positive effect on society and others.  I feel capable of helping the host solve problems.  I believe my suggestions can help the host improve their service.  I feel that by providing suggestions, I can contribute to the host’s improvement and development. |
| P2P accommodation last year  (adapted from Zhang et al., 2025) | How often have you stayed in P2P accommodations during the past year?  1-2  3-4  5-6  More than 7 |

**REFERENCES**

Bove, L. L., Pervan, S. J., Beatty, S. E., & Shiu, E. (2009). Service worker role in encouraging customer organizational citizenship behaviors. *Journal of business research*, *62*(7), 698-705.

Fang, S., Han, X., & Chen, S. (2023). The impact of tourist–robot interaction on tourist engagement in the hospitality industry: A mixed-method study. *Cornell Hospitality Quarterly*, *64*(2), 246-266.

Jaiswal, D., & Kant, R. (2018). Green purchasing behaviour: A conceptual framework and empirical investigation of Indian consumers. *Journal of retailing and consumer services*, *41*, 60-69.

Ren, X., Xia, L., & Du, J. (2018). Delivering warmth by hand: Customer responses to different formats of written communication. *Journal of Services Marketing*, *32*(2), 223-234.

Tang, X., & Fong, L. H. N. (2024). Does perceived yuanfen impact Chinese customers’ hotel ratings?. *International Journal of Hospitality Management*, *122*, 103871.

Zhang, G., Liu, Y., Cheng, M., & Du, M. (2025). While I’m not here, I can still welcome you home: how space–guest interactions shape P2P accommodation appeal. *International Journal of Contemporary Hospitality Management*, *37*(9), 3061-3079.
